# In Vivo Measurement and Characterization of a Novel Formulation of [^177^Lu]-DOTA-Octreotate

**DOI:** 10.7508/aojnmb.2016.04.005

**Published:** 2016

**Authors:** Dale L Bailey, Thomas M Hennessy, Kathy P Willowson, E Courtney Henry, David LH Chan, Alireza Aslani, Paul J Roach

**Affiliations:** 1 Department of Nuclear Medicine, Royal North Shore Hospital, Sydney, Australia; 2 Department of Nuclear Medicine, Prince of Wales Hospital Randwick, Sydney Australia; 3 Institute of Medical Physics, University of Sydney, Sydney, Australia

**Keywords:** Neuroendocrine tumour, Peptide receptor radionuclide therapy (PRRT), Quantification, SPECT

## Abstract

**Objective(s):**

Lutetium-177 can be made with high specific activity and with no other isotopes of lutetium present, referred to as “No Carrier Added” (NCA) ^177^Lu. We have radiolabelled DOTA-conjugated peptide DOTA-(Tyr^3^)-octreotate with NCA ^177^Lu (“NCA-LuTATE”) and used it in nearly 40 therapeutic administrations for subjects with neuroendocrine tumours or meningiomas. In this paper, we report on our initial studies on aspects of the biodistribution and dosimetry of NCA-LuTATE from gamma camera 2D whole body (WB) and quantitative 3D SPECT (qSPECT) ^177^Lu imaging.

**Methods:**

Thirteen patients received 39 NCA-LuTATE injections. Extensive WB planar and qSPECT imaging was acquired at approximately 0.5, 4, 24 and 96 h to permit estimates of clearance and radiation dose estimation using MIRD-based methodology (OLINDA-EXM).

**Results:**

The average amount of NCA-Lutate administered per cycle was 7839±520 MBq. Bi-exponential modelling of whole body clearance showed half lives for the fast & slow components of t_½_=2.1±0.6 h and t_½_=58.1±6.6 h respectively. The average effective dose to kidneys was 3.1±1.0 Gy per cycle. In eight patients completing all treatment cycles the average total dose to kidneys was 11.7±3.6 Gy.

**Conclusions:**

We have shown that NCA-LuTATE has an acceptable radiation safety profile and is a suitable alternative to Carrier-Added ^177^Lu formulations. The fast component of the radiopharmaceutical clearance was closely correlated with baseline renal glomerular filtration rate, and this had an impact on radiation dose to the kidneys. In addition, it has less radioactive waste issues and requires less peptide per treatment.

## Introduction

Currently, there is increasing usage of Lutetium-177 (^177^Lu) as a therapeutic radionuclide for the treatment of metastatic cancer. This is due to a number of factors including beta (β^-^) radiation emission, the co-emission of suitable gamma radiation for external detection with a gamma camera, a prolonged half life of 6.7 days which allows a desirable dose of radiation to be delivered to target tissues, and increasing availability. We have previously shown that quantifying the radioactivity contained in the body can be performed in 2D and 3D with a conventional gamma camera and SPECT/CT (1). In this paper we report aspects of the biodistribution and retention of [^177^Lu]-DOTA-Octreotate (“Lutate”) therapy to treat patients with tumours that exhibit over-expression of somatostatin receptors, using ^177^Lu that was produced by a synthesis method which leads to a “No Carrier Added” (NCA) product. The No Carrier Added product has theoretical advantages of potentially higher specific activity, reduced contribution from long-lived radiocontaminants, and less radiolabelled peptide required for the same given amount of radionuclide therapy administered. As we have studied subjects with progressive disease we have not attempted to calculate formal biodistribution in all of the conventional organs and tissues due to the varying nature and location of the metatstatic lesions, but rather have focused on organs of particular interest for this therapy including those which may prove to be dose-limiting target tissues.

## Materials and Methods

### ^177^Lu and NCA-Lutate Synthesis

The ^177^Lu (t_½_=6.7 days) that has been used in this work was produced in a nuclear reactor by an (n,γ) reaction (Isotope Technologies Garching (ITG) GmbH, Germany and the Australian Nuclear Science & Technology Organisation (ANSTO), Sydney, Australia). Ytterbium-176 (^176^Yb) is irradiated to produce ^177^Yb which decays with a half life of 1.9 hours to ^177^Lu. This method of production produces “No Carrier Added” (NCA) ^177^Lu as the irradiated ytterbium target is readily separated chemically from the lutetium daughter product. An alternative method to make ^177^Lu using the ^176^Lu (n, γ) ^177^Lu reaction will contain other isotopes of lutetium (^176^Lu, ^177m^Lu) as contaminants in the final product. The NCA-LuTATE for injection was produced using DOTA-Octreotate (Auspep Pty Ltd, Melbourne Australia) by the methodology detailed in our previous publication on the subject (2).

### Planar and SPECT/CT Imaging

As reported in a previous publication related to this work (1) we have used two SPECT/CT gamma cameras for all of the imaging used for analysis in this paper (Siemens Symbia. T6 and Intevo, Siemens Healthcare, Hoffman Estates, IL, USA). The protocols for the data acquisition have been documented in the previous report and the reader is referred to this for further information.

Planar whole body (WB) images were acquired after administration of [^177^Lu]-DOTATATE therapy. All patients received an infusion of an amino acid solution for 3 hours to provide renal protection (3), during which time the NCA-Lutate was administered. In the initial cohort, WB planar images were acquired immediately after the end of the NCA-Lutate infusion prior to any losses from the body thus retaining 100% of the administered dose in the body. This approach was used to validate the accuracy of the quantitative planar whole body imaging as previously reported (1). Attenuation correction was applied based on the MIRD Committee approach using the transmission and reference scans with the ^57^Co sheet source (4) to produce quantitative planar images. Imaging at subsequent time points was also quantified using the same method. After collecting a series of WB patient studies using this approach the planar imaging at the initial time point was replaced by a SPECT image. To calculate whole-body retention and clearance rates in this case, referred to as the second cohort, the administered activity measured in the dose calibrator was used as the initial time point. The total radioactivity remaining in the body at each time point was measured from the planar images and fitted with a bi-exponential function to produce two clearance rate constants, representing a fast component and a slow component of clearance. As the radionuclide itself delivers the therapeutic effect via the emitted β^-^ radiation, none of the data that we present are corrected for radionuclide decay. Thus when we refer to half clearance times this includes the component due to radionuclide decay along with the elimination of the radiopharmaceutical from the body.

SPECT/CT imaging has also been acquired in the patients. Images were routinely acquired over the abdomen to include the kidneys as the determination of the radiation absorbed dose to the kidneys was the main purpose of the SPECT imaging. The initial WB planar imaging immediately after administration was replaced after a number of patient studies had been acquired with an early SPECT image along with a venous blood sample, referred to as cohort 2. This was done to examine the accuracy of the quantification (kBq.mL^-1^) of ^177^Lu in vivo, referred to as qSPECT (“quantitative SPECT”), as reported in Bailey et al (1). The complete imaging schedule is shown in Table 1.

**Table 1:** Imaging schedule for each cycle of Lutate therapy

Time point (h)	Procedure	Cohort 1	Cohort 2
Morning of RNT	Reference (“blank”) scan with ^57^Co	•	•
-1	Transmission scan with ^57^Co	•	•
0	Administration of Lutate, ~0-25 min	•	•
0.5	WB planar emission scan (^177^Lu) – cohort 1 OR SPECT/CT scan of abdomen (^177^Lu) – cohort 2	•	
			•
~4	WB planar emission scan (^177^Lu) & SPECT/CT scan of abdomen (^177^Lu)	•	•
~24	WB planar emission scan (^177^Lu) & SPECT/CT scan of abdomen (^177^Lu)	•	•
~96-120	WB planar emission scan (^177^Lu) & SPECT/CT scan of abdomen (^177^Lu)	•	•

Radiation absorbed doses to kidney, liver, bone marrow and spleen were evaluated from qSPECT images using a software package to segment organs and tissues on CT images (DOSIsoft, Caen, France) and the commonly used radionuclide dosimetry software from Vanderbilt University known as OLINDA/EXM (5). OLINDA/EXM (Organ Level Internal Dose Assessment/EXponential Modelling) is a MIRD-based software package designed to estimate radiation absorbed doses from internal radionuclides. The program has found widespread use in calculating doses for radiopharmaceuticals and radionuclide therapeutics, with results comparable to manual calculations of internal dose using its predecessor, MIRDOSE 3.1 (6). The EXM part of the package is used to fit a multi-exponential model to a set of radioactivity measurements to calculate a cumulative radioactivity inside a source organ/region (Ã). A bi-exponential model was fitted to the non decay-corrected data used for all calculations. OLINDA uses a standard male or female phantom to define a patient geometry, and calculates organ doses. Only β^-^ self-doses were considered, neglecting cross-fire doses from other organs and tissues. As the majority of the radiation dose deposition from NCA-Lutate is from β^-^ radiation, with γ-rays providing only a minor contribution (7), this is considered to be a valid assumption.

The segmentation software allows volumes of interest (VOIs) to be defined on a CT scan co-registered with the SPECT images. The software then sums the radioactivity concentration within the segmented region on the SPECT image to give total radioactivity in the VOI. This was repeated for all time points. The radioactivity data, expressed as percentage of injected dose (%ID), were then analysed using OLINDA/EXM to fit a bi-exponential model to the data and find the cumulative activity in each “source organ.”

For kidney and spleen, the %ID values were obtained by manually segmenting the whole organ from the co-registered CT and summing all radioactivity within the organ. The DOSIsoft software then gives an estimate of the CT organ size as well as the qSPECT total radioactivity. As previous publications have demonstrated a large difference in the absorbed dose depending on the organ size (8), the OLINDA model organ sizes were adjusted to the volume measured for each patient from the CT image.

Liver doses were also calculated from the qSPECT images, however, due to liver metastases being common, the entire organ could not easily be segmented into a single, homogeneous VOI. Instead a number of small VOIs (3×10 mm radius spheres) were placed at various locations throughout the liver in areas visually identified as being free of lesions or metastases and the average radioactivity concentration within the VOI measured. This average radioactivity concentration was assumed to be homogenous across the healthy liver, and was scaled to the OLINDA model liver size. Sandstrom reported that this small VOI method could give accurate radiation dose estimates to permit organ dosimetry, with measurements from multiple small liver VOIs differing by <8% (8).

The bone marrow activity concentration was assumed to be equal to the blood concentration as proposed by Forrer (9). For each patient considered, a single blood sample was taken at the midpoint of either the 0.5 or 4 hour SPECT acquisition. The activity concentration was validated by comparison to a standard contained within the field of view, and then compared to the measurement from qSPECT at the same time point. This was used to find a patient-specific correction factor for the qSPECT measurements (this was necessary due to incomplete recovery of counts by the SPECT camera from the small diameter aorta). It was assumed that for an individual patient, this correction factor would stay constant over a treatment cycle. Fitting of a bi-exponential model to the corrected counts showed that qSPECT determined activity was clearing in the expected manner. This activity concentration was then scaled to the OLINDA model’s expected bone marrow volume and cumulative radioactivity determined.

As part of the standard work-up prior to commencing therapy with Lutate, all patients had baseline renal function assessed using [^99m^Tc]-DTPA for glomerular filtration rate (GFR in mL.min^-1^) as well as effective GFR (eGFR) estimated from serum creatinine levels. To allow comparison between patients the GFR was normalised to a standard body surface area of 1.73 m^2^ (i.e., mL.min^-1^.1.73 m^-2^).

## Results

Thirteen patients have been studied after receiving between one and four cycles of NCA-Lutate therapy giving 39 administrations in total. Each cycle can generate up to 4 imaging time points at which planar WB and SPECT imaging may be performed (Table 1). The overall patient summary & demographics are shown in Table 2.

**Table 2:** Lutate therapy patient demographics summary

Characteristic	Value
Total number of therapy patients	13
• Males	8
• Females	5
Age - range	32.6 – 74.7
Age – mean	55.6 yrs
Age - median	60.3 yrs
Weight – mean	71.0 kg
BMI - Mean	24.3 kg.m^-2^
Total number of therapies administered	39
Lutate therapy amount administered - mean	7839 MBq

### Whole Body Retention

**Figure 1:**
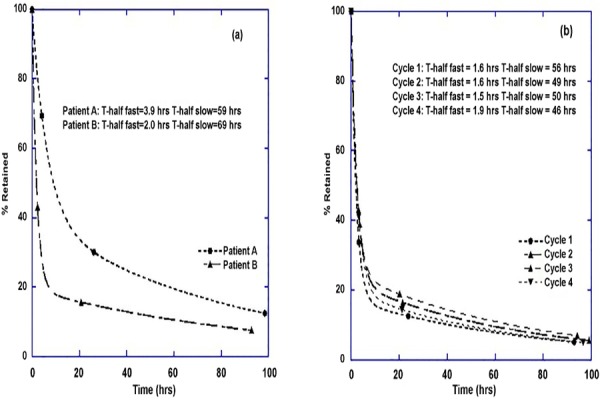
Example clearance curves are shown. In figure (a) two different patients are with very different clearance profiles are compared. Their baseline GFR measurements were 65 mL.min^-1^.1.73m^-2^ (Patient A) and 91 mL.min^-1^.1.73m^-2^ (Patient B). In figure (b) the clearance profiles from 4 cycles in the same patient are shown. The high degree of reproducibility seen here is common

Whole body retention and clearance rates were assessed in 36 individual administrations in thirteen patients, the remaining 3 not being able to complete the entire imaging protocol. Examples of retention curves are shown in Figure 1. For the 36 patient biodistributions studied the mean clearance half times measured were 2.1±0.6 h (range: 1.4 - 3.9 h) for the fast component and 58.1±6.6 h (range: 46.0 – 70.0 h) for the slow component.

**Figure 2:**
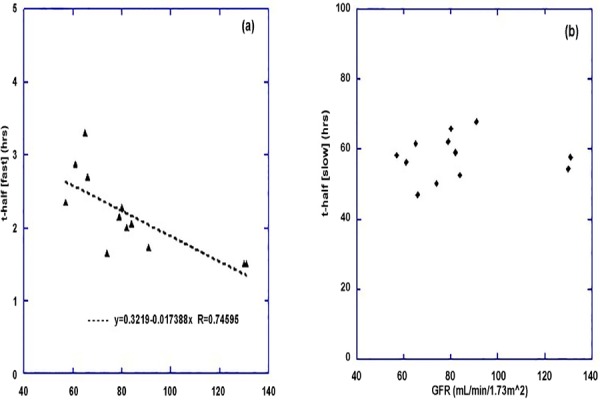
(a) The fast component of whole body clearance as determined from the gamma camera planar whole body measurements is shown as a function of the baseline GFR measurement for 12 subjects. The value of the fast component shown is the mean of all measurements for each individual. A similar plot for the slow component of whole body clearance (b) showed no correlation with baseline GFR

We examined the relationship between the baseline normalised GFR and the fast component of whole body clearance for each individual. An inverse correlation was observed such that the fast component of clearance decreased by 1.74 × 10^-2^ h (approx. 1 min) per mL.min^-1^ increase in normalised GFR, with a correlation coefficient of 0.75 (Figure 2a) and which was statistically significant (P=0.0053). No such dependence was observed when comparing the slow component to GFR (Figure 2b).

### Dosimetry to Kidneys and Other Tissues

The absorbed dose to the kidneys as determined by the qSPECT imaging and OLINDA/EXM using a bi-exponential fit yielded a mean dose per cycle of 3.1±1.0 Gy (range: 1.1 – 5.4 Gy; n=36) or 0.40±0.13 mGy.MBq^-1^ (range: 0.12 – 0.67 mGy.MBq^-1^). The dose to bone marrow was estimated in eight patients where contemporaneous venous blood samples were obtained during the qSPECT image acquisition. Doses to liver and spleen were also estimated using the methods described previously. All results are shown in Table 3.

**Table 3:** Dose Estimates from nca-Lutate to Organs from qSPECT imaging

Organ	Mean Total Dose per cycle (Gy)	Dose/Unit Radioactivity (mGy/MBq)	Number of Observations
Kidneys	3.1 ± 1.0	0.40 ± 0.13	36
Red Marrow	0.086 ± 0.020	0.011 ± 0.002	8
Liver	0.56 ± 0.21	0.071 ± 0.027	35
Spleen	3.6 ± 1.5	0.46 ± 0.20	36

## Discussion

This paper represents the third contribution from our group detailing the introduction of a new formulation of [^177^Lu]-DOTA-Octreotate. The previous publications documented the synthesis of the NCA-Lutate (2) and the quantitative imaging of ^177^Lu with the gamma camera (1). The Lutate that we have used in this paper differs in two respects from that used in other publications: (i) it uses a different approach to the production of ^177^Lu that results in a NCA radionuclide, and, (ii) this has been conjugated to a locally-produced octreotate peptide. The NCA characteristic of this product has favourable properties such as the lack of other long-lived isotopes of lutetium which may require long-term storage to allow for decay to acceptable levels prior to disposal. The current paper utilises the methodology developed in the first two papers mentioned to report on aspects of the pharmacokinetics and radiation dosimetry of this new form of Lutate.

We have used a comprehensive imaging protocol over a number of days after administration of the NCA-Lutate in the majority of our patients to provide robust numbers for the analysis. We have observed that the pattern of elimination of the NCA-Lutate from the body as measured from the whole-body planar images is highly reproducible within an individual. By having 3-4 measurement time points we were able to fit a multi-exponential clearance curve to better characterise the clearance profile than a mono-exponential curve is able to do. While this protocol requires a high degree of cooperation from the patients, we have found the majority willing to participate in order to help us to understand the process more completely. Being able to obtain serial data after the administration of the therapy provides knowledge in areas such as radiation safety/protection for the time after the Lutate administration when it is usually permissible to allow the patient to resume contact with members of the general public. It also helps in identifying factors affecting retention and the optimal timing of ^177^Lu imaging to monitor disease progression using the therapeutic injection.

Our results indicate that the estimated radiation doses to the kidneys, bone marrow, liver and spleen are, generally, comparable with or slightly lower than those previously reported. Table 4 shows a comparison from a number of publications of the estimated radiation dosimetry from [^177^Lu]-labelled somatostatin receptor radionuclide therapy.

**Table 4:** Comparison of Published Radiation Dose Estimates for Different Formulations of [^177^Lu]-DOTA-Octreotate (3, 7-11)

Organ	Dose (mGy/MBq)	No. of Subjects	Reference
Kidney	1.65 ± 0.47 (no renal protection) 0.88 ± 0.19 (with renal protection)	6 5	Kwekkeboom (2001) Kwekkeboom (2001)
	0.62 (0.45 – 17.74)	10	Cremonesi (2006)
	0.9 ± 0.3	89	Wehrmann (2007)
	(0.32 – 1.67)	24	Sandstrom (2010)
	(2 -10 Gy for 7.4GBq)	200	Sandstrom (2013)
	0.40 ± 0.13	36	This paper
Red Marrow	0.07 ± 0.01	6	Kwekkeboom (2001)
	0.04 (0.02 – 0.06)	10	Cremonesi (2006)
	0.04 ± 0.02	27	Wehrmann (2007)
	0.02 ± 0.03	14	Forrer (2009)
	(0.05 - 0.4 Gy for 7.4GBq)	200	Sandstrom (2013)
	0.011 ± 0.002	8	This paper
Liver	0.18 (0.05 – 0.34)	10	Cremonesi (2006)
	(0.13 – 1.10)	24	Sandstrom (2010)
	0.21 ± 0.08	6	Kwekkeboom (2001)
	0.071 ± 0.027	35	This paper
Spleen	2.15 ± 0.39	6	Kwekkeboom (2001)
	1.2 ± 0.5	73	Wehrmann (2007)
	0.64 (0.29-2.91)	10	Cremonesi (2006)
	0.21 – 1.86	24	Sandstrom (2010)
	0.46 ± 0.20	36	This paper

Our results are similar to those recently reported by Limouris et al for their formulation of NCA-Lutate, although the results are difficult to directly compare as they administered their NCA-Lutate via the hepatic artery as an intra-arterial injection (12). A number of favourable aspects of our protocol may contribute to the equivalent or lower estimates of the dosimetry seen in this series compared to those using carrier-added Lutate including:

- the comprehensive imaging protocol allowing a better fit to be applied to the image-derived data;
- a new formulation of the peptide not previously reported;
- the use of amino acid protection to prevent reabsorption of the compound in the kidneys thereby hastening the transit of the Lutate through the renal tissues;
- a novel form of ^177^Lu which is carrier free and contains no other isotopes of lutetium;
- generally, a large number of samples were analysed (apart from the red bone marrow/blood, which consisted of only 8 data points).

**Figure 3:**
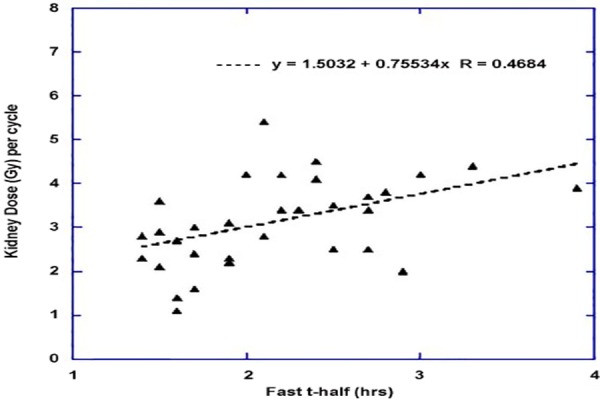
Relationship between the fast component of whole body clearance and the calculated radiation absorbed dose to the kidneys is shown

The image data that we have collected has been included in a database (REDCap, Vanderbilt University, USA) along with a large number of other parameters related to the patient including demographics, histopathology, quality-of-life assessments, performance status, previous medical history and treatment, biochemistry and blood results. This has allowed us to explore correlates with that which has been observed in the imaging. One of our first observations is that the fast component of the whole body clearance profile shows a strong dependence on the baseline GFR (Figure 2a). This is perhaps not surprising for an agent which is rapidly cleared by the kidneys. To explore this further we have looked at individual estimates of the radiation dose absorbed by the kidneys as a function of the fast component of clearance for each administration. The relationship is shown in Figure 3.

As can be seen in the figure, the slower the clearance from the body the higher the radiation dose delivered to the kidneys. As the kidney is regarded as an organ at high risk in [^177^Lu]-labelled somatostatin receptor radionuclide therapy that implies consideration of patient’s baseline GFR, if using a personalised approach to prescribing the amount of Lutate required. While the issue of renal toxicity from Lutate therapy is still debatable, nevertheless, a patient with a poor baseline GFR would appear to be able to be given a lesser amount of Lutate before reaching a threshold radiation dose to kidneys compared with someone with much higher baseline GFR. Conversely, the patient with a good baseline GFR could be given even larger amounts of Lutate with the intention of delivering greater radiation dose to the tumours while staying below the threshold for the kidneys.

## Conclusion

We have used an extensive imaging protocol after the administration of DOTA-(Tyr^3^)-octreotate radiolabelled with no carrier added ^177^Lu to characterise the in vivo kinetics. It has been shown to have a favourable biodistribution and has been estimated to give a tolerable radiation dose to the kidneys and other critical organs. We are currently collecting data on the clinical efficacy of this therapeutic compound. It would appear to be a safe and effective modality for treating patients with somatostatin expressing cancers.

## Funding

This work was funded, in part, by an educational grant provided by the Australian Nuclear Science & Technology Organisation (ANSTO, Sydney, Australia).

The Neuro-Endocrine Tumour project at RNSH (NETwork) is partly funded by Sydney Vital through the Translational Cancer Research Centre programme of the Cancer Institute NSW.

## Conflict of Interest

DLB receives research support from Sydney Vital through the Translational Cancer Research Centre programme of the Cancer Institute NSW.

TMH was supported for part of this work by an educational grant provided by the Australian Nuclear Science & Technology Organisation (ANSTO, Lucas Hts, Australia).

KPW is a Research Fellow of the University of Sydney supported by a Linkage Project grant from the Australian Research Council (ARC) in partnership with Sirtex Medical Pty Ltd (Sydney, Australia).

DLHC is a Clinical Research Fellow funded by Sydney Vital through the Translational Cancer Research Centre programme of the Cancer Institute NSW.

ECH, AA and PJR have no conflicts of interest in regard to this work.

### Ethical Approval

All procedures performed in studies involving human participants were in accordance with the ethical standards of the institutional and/or national research committee and with the 1964 Helsinki declaration and its later amendments or comparable ethical standards.

## Authors’ Contributions

DLB conceived the study, collaborated on the design of the protocols, developed the analysis software where required and wrote the initial draft of the manuscript. TMH carried out much of the data analysis of the planar whole body imaging studies and analysed the results and revised the manuscript. KPW developed the quantitative SPECT methodology and implemented it for ^177^Lu on the gamma cameras and revised the manuscript. ECH performed the bulk of the data analysis for the quantitative SPECT studies and analysed the results and revised the manuscript. DLHC provided the clinical details about the subjects and revised the in vivo experimental results and revised the manuscript. AA performed all of the blood-based data collection and analysis and the comparison of the ex vivo and in vivo blood sampling results and revised the manuscript. PJR provided input into the design of the protocols, subject recruitment, analysis of data, manuscript development and revision.

All authors read and approved the final manuscript.
